# The effect of pre-analytical treatment on the results of stoichiometric measurements in invertebrates

**DOI:** 10.1007/s13355-015-0346-7

**Published:** 2015-05-26

**Authors:** Anna Rożen, Łukasz Sobczyk, January Weiner

**Affiliations:** Institute of Environmental Sciences, Jagiellonian University, ul. Gronostajowa 7, 30-387 Kraków, Poland

**Keywords:** Insects, Earthworms, Pitfall traps, Preservation, Elemental body composition

## Abstract

Growing interest in the application of stoichiometric approaches to community ecology has resulted in an increasing number of studies examining invertebrate body composition. Our experiments demonstrate various sources of possible error related to the use of pre-analytical procedures. We examined the effects of different preservatives (ethanol and formaldehyde) used in pitfall traps, time of preservation (2 weeks or 3 days) and drying method (vacuum drying at 50 °C and freeze-drying) on the determination of body composition in invertebrates representing taxa often used in such studies: earthworms and five species of insects (adults or larvae). The contents of C, N, S, P, Fe, Zn, Cu, Mn, Ca, Mg and K in each animal were measured. The use of solvents (ethanol or formaldehyde) in pitfall traps and for preservation significantly affects the body composition and stoichiometry of earthworms, even during short exposure times. Insects (both adults and larvae) were affected only during a 2-week exposure; 3 days of exposure did not significantly change their chemical composition. Vacuum-oven drying of animals at 50 °C does not affect their body composition relative to freeze-drying.

## Introduction

Growing interest in the application of stoichiometric approaches to community ecology has resulted in numerous studies examining the body composition of invertebrates (Bertram et al. 2008; González et al. 2011; Pokarzhevskii et al. 2003; Woods et al. 2004). Another emerging area of interest is ecotoxicology: the precise determination of the concentration of elements (especially heavy metals) in animal bodies. Invertebrates are used to test toxic substances and serve as indicators of pollution in the field (Spurgeon et al. 2003).

Analytical procedures are not error-free; each procedure can be riddled with errors ranging from the more preventable (e.g., imprecise execution of a procedure) to the ones difficult to prevent (e.g., equipment failures). Nevertheless, these errors can often be detected and avoided through the use of certified materials (with known elemental concentration) as controls. However, the measured element content can also be affected by the way in which study animals are treated pre-analytically. Such differences are a source of systematic error. In ecological stoichiometry, the body contents of several elements must be measured in numerous individuals, particularly if small-bodied organisms, such as soil and litter invertebrates, are studied, and this requires extensive sampling in the field. The accepted way to sample animals for analytical purposes is to catch them alive and kill them immediately by freezing and drying (lyophilization; Bertram et al. 2008; Cross et al. 2003; Marichal et al. 2011; Woods et al. 2004). For practical reasons, such a procedure is not always possible. A commonly used method of sampling litter invertebrates is pitfall trapping (Duelli et al. 1999). This method allows the composition of macrofaunal communities to be screened so that a diverse number of individuals from different taxa are sampled without disturbing the environment. Pitfall traps can be exposed for various time periods, from 1 day to several weeks (Duelli et al. 1999). Pitfall traps are most commonly filled with ethanol, ethylene glycol (automobile antifreeze) or formaldehyde solution (Braun et al. 2009; Gibb and Oseto 2006; Knapp 2012). These chemicals prevent animals from escaping, kill them quickly and effectively preserve them for further investigations. However, preservation can also affect the chemical body composition—some elements can be washed out as a result of the partial dehydration of organisms; nevertheless, some authors continue to use ethanol to kill animals for elemental analysis (e.g., Nakamura et al. 2005). Among numerous papers on the impact of pitfall traps on collected animals, only a few address this problem. Zödl and Wittmann (2003) studied the effects of pitfall traps on metal concentrations in selected invertebrates. Braun et al. (2009), 2012 studied the consequences of the composition and concentration of reagents used in pitfall traps and the preservation of firebugs (*Pyrrhocoris apterus* Fallén) on elemental analysis, while Knapp (2012) investigated the impact of preservative fluid and storage conditions on the estimation of body mass in carabid beetles [*Anchomenus dorsalis* (Pontoppidan)].

Prior to elemental analyses, samples have to be dried. Drying can take place at high temperatures in standard driers, vacuum driers (which allow samples to dry at lower temperatures than standard ones) or by dehydration at low temperatures (freeze-drying; Couture et al. 2010; Cross et al. 2003). The most commonly used temperatures for drying animals are 60 °C and 50 °C (Bertram et al. 2006, 2008; Nakamura et al. 2005; Nakamura and Taira 2005; Kagata and Ohgushi 2007; Kay et al. 2006; Larsen et al. 2009, 2011; Marichal et al. 2011; Villanueva et al. 2011; Woods et al. 2004). Numerous papers that report on the chemical body composition of invertebrates do not provide complete information on the sampling and analytical prepping procedures used (Hambäck et al. 2009). The drying procedure may change elemental composition (e.g., due to the vaporization of some compounds). Preservation methods differ in their effects on particular invertebrate taxa and developmental stages (e.g., soft-bodied, high water content animals, such as annelid worms, and chitinized arthropods, such as beetle imagines).

The goal of our experiments was to identify sources of error resulting from pre-analytical procedures in physiologically different species of invertebrates. We employed commonly used, commercially available animals for our analysis, including earthworms, representing an important and abundant group of soil animals used in ecotoxicological tests, and various insect species at the adult and larval stages. Animals differed in their sensitivity to desiccation, water and fat content and their degree of chitinization (Finke 2002, 2007; Finke and Winn 2004).

We tested two killing agents routinely used in the traps: ethanol and formaldehyde. We excluded ethylene glycol from the study because this agent forms a sticky layer on the animals’ body that may affect both body mass and composition, especially when it is used in the form of automobile antifreeze (Braun et al. 2009, 2012; Knapp 2012). This property, therefore, makes use of ethylene glycol unsuitable for preserving animals for chemical analysis.

The aims of the study were to answer the following questions:

1. How do different preservation methods influence the concentrations of C, N, S, P, Fe, Ca, Mg, Mn, K, Zn and Cu in invertebrates?
2. How do the effects observed in soft-bodied invertebrates (i.e., earthworms) differ from those observed in invertebrates with chitinized bodies (e.g., insects)?
3. How does the time of preservation affect the body composition of invertebrates?
4. How do drying methods affect the determination of invertebrate body dry mass and composition?

## Materials and methods

### Experimental animals

The following species of invertebrates were used for this study: earthworms *Dendrobaena veneta* (Rosa) (Oligochaeta: Lumbricidae), crickets *Gryllus assimilis* (Fabricius) (Orthoptera: Gryllidae), two species of tenebrionid larvae: *Tenebrio molitor* L. (Coleoptera: Tenebrionidae) and *Zophobas morio* Fabricius (Coleoptera: Tenebrionidae), house fly larvae (*Musca domestica* L., Diptera: Muscidae) and Guyana orange spotted cockroach (*Blaptica dubia* Serville, Blattodea: Blaberidae). The earthworms were subdivided into two size classes: small (immature, smaller than 0.018 g dry mass) and large (subadult and adult heavier than 0.04 g dry mass). All animals originated from commercial cultures.

### Experiment description

To assess the impact of different preservatives (ethanol and formaldehyde) on invertebrate body composition, the impact of preservation time and the impact of the drying method on the determination of the body composition of invertebrates, we conducted three experiments:

#### Long exposure simulation (2 weeks of pitfall trap exposure)

Each of the above-listed animal species was divided into three groups treated in the following ways:

- group 1 (reference): frozen (in −20 °C, after taking from culture), to determine the body composition of untreated animals (*N* = 5);
- group 2: stored in 4 % formaldehyde for 2 weeks (*N* = 5);
- group 3: stored in 70 % ethanol for 2 weeks (*N* = 5).

Before the experiment, the earthworms were kept on moist blotting paper for 48 h to clean the gut. Before weighing, the animals preserved in ethanol or formaldehyde were gently dried on blotting paper. Thereafter, the animals were dried in a vacuum drier at 50 °C for 48 h.

#### Short exposure simulation

Based on the results of the previous experiment, the short exposure simulation was performed on selected groups of animals. Only adult *D. veneta*, the species most strongly influenced by preservation methods, and *M. domestica* larvae, the species least influenced by preservation methods, were used in this experiment as representative insects. Animals were treated as in the previous experiment, but only for 3 days: (1) frozen (*N* = 9), (2) stored in 4 % formaldehyde (*N* = 9) and (3) stored in 70 % ethanol (*N* = 9). Earthworms were kept before the experiment for 48 h on moist blotting paper to clean the gut. After treatment, the animals were dried in a vacuum drier at 50 °C for 48 h.

#### Drying method simulation

Again, only earthworms (*N* = 5) and house fly larvae (*N* = 8) were used. The animals were treated as described for the short exposure simulation, then subdivided into two groups: one dried in a vacuum drier at 50 °C for 48 h and the other freeze-dried for 7 days.

### Analytical procedures

Dried animals were powdered in a porcelain mortar before analysis. Because body size differs among the species studied, different numbers of individuals were taken per sample: one (earthworms, *Zophobas* larvae, cockroaches, crickets), two (fly larvae) or three (*Trenebrio* larvae), so that the total sample mass (not lower than 0.1 g dry mass) would allow all studied elements to be analyzed in one individual or in one sample. The contents of C, N, S, Fe, Zn, Cu, Mn, Ca, Mg and K were measured in the long-exposure simulation, and P, Fe, Zn, Cu, Mn, Ca and Mg were analyzed in the short-exposure simulation.

Total C and total N contents were determined using the Perkin-Elmer CHN analyzer. The metal content was determined by atomic absorption. Dried samples were digested in 5 ml boiling, concentrated (65 %) nitric acid (Suprapur, Merck). When the fumes were white and the solution was completely clear, the sample evaporated, cooled to room temperature and was filled with up to 30 ml of deionized water. The digested samples were analyzed for Zn, Fe, Ca, Mgand K by flame AAS (Perkin-Elmer AAnalyst 800) and for Mn and Cu by graphite furnace AAS (Perkin-Elmer AAnalyst 800). Five blank samples of nitric acid accompanied every analytical run.

Phosphorus concentrations were determined in nitric acid digested samples by the colorimetric method using a flow injection analyzer (FIA-System MLE GmbH).

The analytical precision of all analyses was confirmed against certified standard material (NCS ZC81001, pork liver; NCS ZC73016, chicken).

### Statistical analysis

For comparisons of the body composition between taxa (only frozen animals), we used a one-way ANOVA separately for each micro- and macroelement. If the data did not meet normality and homogeneity of variance (C:N, C:S), we used a nonparametric test (Kruskall-Wallis). To compare the elemental concentrations in animals preserved using various methods, we used a one-way ANOVA separately for each taxon, preservation time and element. For short-term preservation, only *D. veneta* and *M. domestica* larvae were used. Pooling together two or three individuals in one sample may mask the individual variation of the results, but at any statistical comparisons of averages this effect is compensated by the reduced number of the degrees of freedom (the confidence limits of the means remain unaffected).

To analyze the differences between groups of samples preserved using three methods and two exposition times with regard to all analyzed elements, we performed a principal component analysis (PCA) on the correlation matrices. We only used the data for earthworms and fly larvae and only the elements analyzed in both long- and short-term exposure times (i.e., only microelements). We then conducted a two-way ANOVA on the scores of the first and second principal component axes to test for significant differences between exposure time and preservation method and to test for interaction effects between exposure time and preservation method.

The effect of drying and preservation method on body mass loss was analyzed only for earthworms and fly larvae. We compared the initial fresh body masses of the experimental animals using a one-way ANOVA. The differences in the loss of fresh body mass during 2 weeks of preservation between animals preserved in ethanol and in formaldehyde were analyzed using a *t* test. To analyze the effect of both preservation and drying method on body mass loss, a two-way ANOVA was used. Results are presented as mean ± SE.

Statistical analyses were performed using the statistical packages STATISTICA 10 and CANOCO 5 (Ter Braak and Šmilauer 2012).

## Results

### Body composition and stoichiometric relations of experimental animals

Only frozen individuals (control samples) were used for comparisons of body composition between taxa.

#### Macroelements

The animals studied differed in both concentrations of macroelements and their stoichiometric relations (Table 1). Earthworm (*D. veneta*) tissues were richer in nitrogen than all other animals studied, whereas the lowest concentration of nitrogen was found in *Z. morio* larvae. *Z. morio* larvae differed clearly from all other animals in carbon content. The concentration of sulfur was the highest in earthworms. C:N and C:S ratios were the lowest in earthworms and the highest in *Z. morio* larvae and *T. molitor* larvae (Table 1).

**Table 1:** Macroelement content (% dry mass ± SE) and C:N, C:S and N:S ratio (molar) in the bodies of studied animals

Species	N (%)	C (%)	S (%)	C:N	C:S*	N:S*
*D. veneta* imm.	11.47 ± 0.20^a^	47.91 ± 0.65^a,d^	0.91 ± 0.09^a^	4.87 ± 0.13^a^	63.15 ± 5.41^a^	29.79 ± 3.21^a^
*D. veneta* ad.	11.33 ± 0.13^a^	48.65 ± 0.09^a,d^	0.80 ± 0.04^a^	5.01 ± 0.06^a^	71.30 ± 3.95^a^	32.49 ± 1.56
*Zophobas morio* larvae	7.58 ± 0.33^b^	58.17 ± 0.62^b,e^	0.56 ± 0.07^b,c^	8.98 ± 0.33^b^	121.82 ± 10.77^b^	31.20 ± 3.05^a^
*Tenebrio molitor* larvae	9.14 ± 0.26^c,d^	50.14 ± 0.84^a,c,d^	0.31 ± 0.02^b^	6.42 ± 0.21^c,d^	193.83 ± 15.79^b^	68.63 ± 3.68^b^
*Blaptica dubia*	9.34 ± 0.11^c,d^	52.05 ± 1.08^a,c^	0.47 ± 0.11^b^	6.51 ± 0.15^c,d^	160.94 ± 34.54	55.93 ± 11.60
*Gryllus assimilis*	9.78 ± 0.21^d^	48.03 ± 0.98^d^	0.48 ± 0.01^b^	5.74 ± 0.18^a,d^	117.48 ± 1.98	46.94 ± 1.61
*Musca domestica* larvae	8.47 ± 0.14^b,c^	52.69 ± 1.14^c,e^	0.51 ± 0.03^b,c^	7.25 ± 0.17^c^	119.26 ± 6.29	37.65 ± 2.13

Superscript letters denote homogeneous groups (one-way ANOVA, Tukey’s HSD test *p* < 0.05, or * Kruskal-Wallis test, and post hoc multiple comparison by ranks *p* < 0.05)

#### Microelements

Some microelements differed significantly in their concentrations in the species studied (Table 2). The earthworms contained much higher concentrations of iron and calcium, *T. molitor* larvae contained higher concentrations of magnesium, and *T. molitor* and *G. assimilis* contained higher concentrations of copper than any other animals studied here (Table 2).

**Table 2:** Microelement content in the bodies of animals studied (mg kg^−1^ dry mass ± SE)

Species	Fe (mg kg^−1^)	Zn (mg kg^−1^)	Mn (mg kg^−1^)	Cu (mg kg^−1^)	Ca (mg kg^−1^)	Mg (mg kg^−1^)	K (mg kg^−1^)
*D. veneta* imm.	103.27 ± 15.17^a^	140.53 ± 27.94	5.03 ± 1.60^a^	7.60 ± 0.53^a^	5249.68 ± 343.73^a^	879.01 ± 141.83^a^	8547.90 ± 457.07^a^
*D. veneta* ad.	247.45 ± 56.41^b^	110.04 ± 2.15	10.06 ± 4.06^a,c^	7.34 ± 0.86^a^	3075.89 ± 144.13^b,d^	712.83 ± 12.97^a^	7230.46 ± 176.69^a^
*Zophobas morio* larvae	62.06 ± 7.83^a^	85.48 ± 5.43^a^	29.85 ± 5.43^a,c^	10.99 ± 1.62^a^	969.39 ± 228.86^c^	1510.84 ± 231.71^a,c^	8231.36 ± 596.05^a^
*Tenebrio molitor* larvae	62.45 ± 9.31^c^	165.53 ± 13.27	16.55 ± 2.89^a,c^	24.39 ± 2.22^b,c^	959.58 ± 107.93^c^	3405.13 ± 146.90^b^	11894.82 ± 825.90^a,c^
*Blaptica dubia*	54.15 ± 4.37^c^	169.15 ± 22.81	36.12 ± 7.24^b,c^	11.77 ± 1.93^a^	643.25 ± 132.72^c^	1898.75 ± 204.67^c^	10089.69 ± 1336.48^a^
*Gryllus assimilis*	83.94 ± 6.12^a^	201.73 ± 21.10^b^	58.13 ± 7.06^b^	25.12 ± 4.22^b^	2261.76 ± 259.59^b,d^	966.91 ± 39.37^a^	14769.57 ± 599.48^b,c^
*Musca domestica* larvae	70.50 ± 5.31^a^	106.23 ± 6.55^a^	10.44 ± 2.01^a^	11.34 ± 2.00^a,c^	1898.12 ± 381.45^c,d^	846.16 ± 47.05^a^	9631.33 ± 95.129^a^

Superscript letters denote homogenous groups (one-way ANOVA, Tukey’s HSD test *p* < 0.05)

### Impact of preservation methods and time of preservation on body composition

#### Long exposure simulation

##### *Macroelements*

**Fig. 1:**
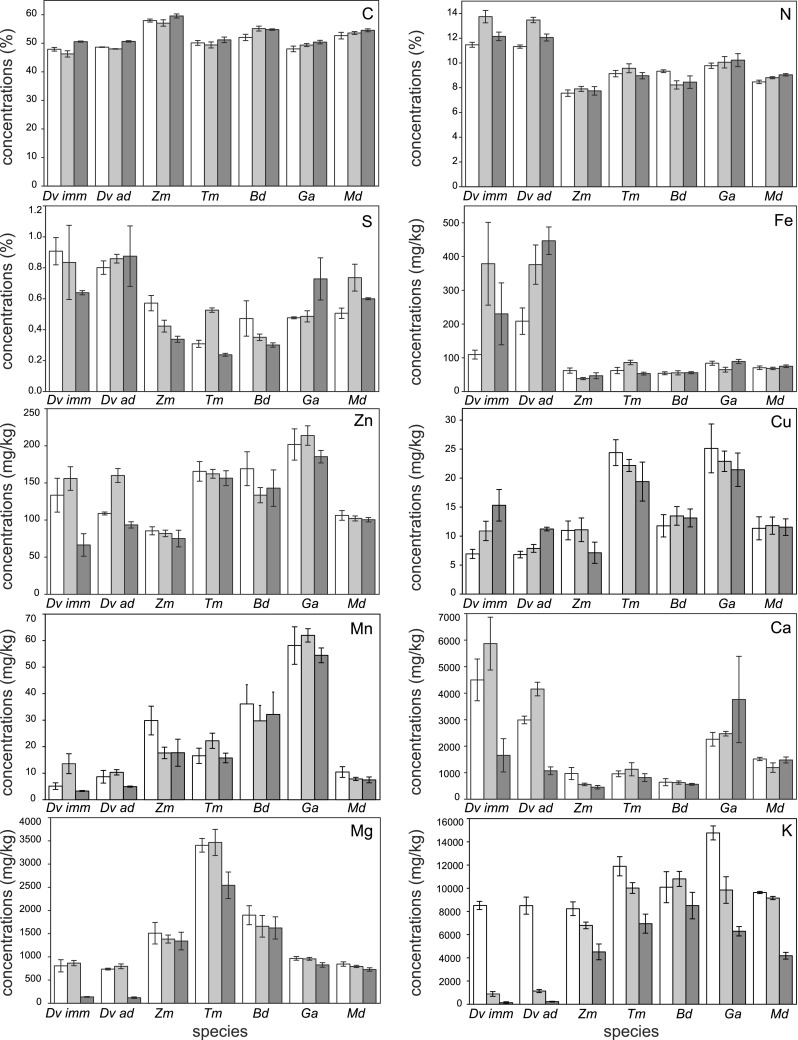
Concentrations of elements (% or mg kg^−1^ dry mass, mean ± SE) in invertebrates that were frozen (*white bars*), preserved in ethanol (*gray bars*) and preserved in formaldehyde (*dark gray bars*) for 2 weeks (long exposure). *Dv*_*imm*_ *Dendrobaena veneta* immature individuals, *Dv*_*ad*_ *Dendrobaena veneta* adult individuals, *Zm* *Zophobas morio* larvae, *Tm* *Tenebrio molitor* larvae, *Bd* *Blaptica dubia*, *Ga* *Gryllus assimilis*, *Md* *Musca domestica* larvae

The preservation method affected the measurement of macroelement content in animal bodies and their stoichiometric relations (Fig. 1, results of ANOVA in Table 3).

**Table 3:** ANOVA table for differences between elemental concentrations in animals preserved using various methods: frozen (**M**), preserved in ethanol (**E**) and in formaldehyde (**F**)

Species	C	N	S	Fe	Zn	Cu	Mn	Ca	Mg	K
Long-term exposition simulation										
*Dendrobaena veneta* imm.	**0.006**	**0.005**	0.48	0.07	**0.04**	**0.02**	0.05	**0.03**	**0.007**	**<0.0001**
	**E** **≠** **F**	**(M, F)** **≠** **E**			**E** **≠** **F**	**M** **≠** **F**		**E** **≠** **F**	**M** **≠** **F**	**M** **≠** **(E, F)**
*D. veneta* adult	**<0.0001**	**0.0009**	0.94	**0.008**	**<0.0001**	**0.003**	0.07	**<0.0001**	**<0.0001**	**<0.0001**
	**(M, E)** **≠** **F**	**(M, F)** **≠** **E**		**M** **≠** **F**	**(M, F)** **≠** **E**	**(M, E)** **≠** **F**		**M** **≠** **E** **≠** **F**	**(M, E)** **≠** **F**	**M** **≠** **(E, F)**
*Zophobas morio* larvae	0.18	0.64	**0.006**	0.09	0.62	0.36	0.12	0.05	0.78	**0.002**
			**M** **≠** **(E, F)**							**M** **≠** **E, F**
*Tenebrio molitor* larvae	0.44	0.36	**<0.0001**	**0.01**	0.80	0.39	0.18	0.49	0.05	**0.002**
			**M** **≠** **E** **≠** **F**	**E** **≠** **F**						**M** **≠** **E, F**
*Blaptica dubia*	**0.04**	0.1	0.35	0.96	0.46	0.77	0.82	0.80	0.66	0.16
	**M** **≠** **E**									
*Gryllus assimilis*	0.12	0.75	0.08	0.06	0.44	0.71	0.53	0.50	0.05	**<0.0001**
										**M** **≠** **E** **≠** **F**
*Musca domestica* larvae	0.3	**0.02**	0.06	0.53	0.65	0.98	0.26	0.26	0.11	**<0.0001**
		**M** **≠** **F**								**M** **≠** **E, F**
Short-term exposition simulation										
*D. veneta* adult				**0.01**	**0.0004**	0.13	0.92	**0.013**	**<0.0001**	**<0.0001**
				**M** **≠** **E**	**(M, F)** **≠** **E**			**E** **≠** **F**	**(M, E)** **≠** **F**	**M** **≠** **E** **≠** **F**
*M. domestica* larvae				0.14	0.85	0.32	0.1	0.08	**0.016**	0.48
									**M** **≠** **E**	

Values that indicate significance probability levels (*p*) are given. For *p* < 0.05, the results of Tukey’s HSD post hoc tests of differences between treatments are given (≠ denotes a significant difference between the groups indicated)

For earthworms, significant differences were found in carbon (higher concentration in formaldehyde preserved individuals) and nitrogen (higher concentration in ethanol preserved individuals). For insects, statistically significant differences between samples preserved in different ways were observed for carbon content (*B. dubia*, higher concentration in both ethanol- and formaldehyde-preserved individuals), nitrogen content (*M. domestica* larvae, higher concentration in both ethanol and formaldehyde) and sulfur content (*T. molitor* larvae, higher concentration in ethanol; *M. domestica* larvae, higher concentration in ethanol).

Significant differences in stoichiometric relations were found only in earthworms (C:N ratios lower in ethanol).

##### *Microelements*

**Fig. 2:**
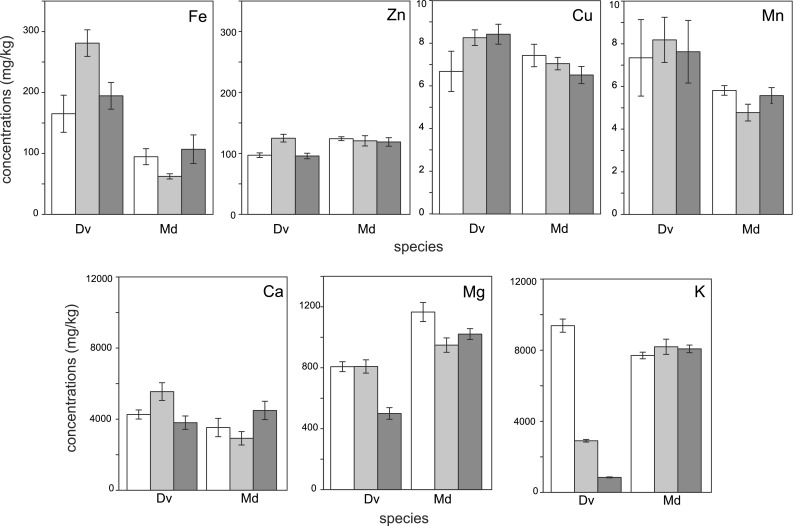
Concentrations of elements (mg kg^−1^ dry mass, mean ± SE) in earthworms (*D. veneta*) and fly larvae (*M. domestica*) preserved for 3 days (short exposure). *White bars* frozen individuals; *gray bars* preserved in ethanol; *dark gray bars* preserved in formaldehyde

Microelement contents in the earthworms were strongly affected by the preservation method (ANOVA results in Table 3). Significant differences were observed in all elements except manganese (Table 3). Potassium was the most easily washed out during preservation of all animals studied (except *B. dubia*) (Fig. 2). Statistically significant differences between preservation methods were also found for iron in *T. molitor* larvae (Fig. 2); however, the difference was only significant between animals preserved in ethanol and formaldehyde.

#### Short exposure simulation

##### *Macroelements*

In this simulation, only the phosphorus concentrations were measured.

In *D. veneta*, the phosphorus concentrations differed significantly in particular treatment groups (*F*_2, 11_ = 36.72, *p* = 0.00001): in frozen animals, the phosphorus concentration was 0.69 % ± 0.03 and was significantly lower both in ethanol (0.41 % ± 0.03) and formaldehyde (0.44 % ± 0.02).

In *M. domestica* larvae, the phosphorus concentration in all treatment groups was similar (0.74 % ± 0.01—frozen, 0.72 % ± 0.01—in ethanol, 0.74 % ± 0.01 in formaldehyde).

##### *Microelements*

The results of microelement analyses in *D. veneta* are similar to those in the long-exposure simulation (Fig. 2). Significant differences were found between treatment groups in all elements except manganese and copper (Table 3).

In *M. domestica* larvae (Fig. 2), a significant difference was found only in magnesium content between ethanol-exposed and frozen individuals; however, such effects were not found in the long-term preservation experiment (Table 3).

### Preservation time

**Fig. 3:**
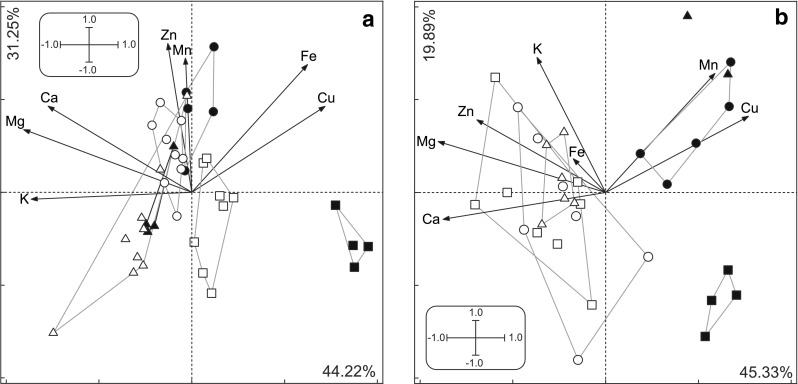
The effect of exposure time (2 weeks vs. 3 days) of various preservation agents on body composition (PCA plot, all elements except C, N, S). **a** earthworms, **b** fly larvae. *Triangles* frozen, *circles* ethanol preserved, *squares* formaldehyde preserved. *Filled symbols* long (2 weeks) exposition, *empty* short (3 days) exposition. Component loadings: **a** *Axis1* Mg = −0.91, K = −0.87, Ca = −0.78, Cu = 0.72, Fe = 0.62, Zn = −0.13, Mn = −0.03. *Axis2* Zn = 0.80, Mn = 0.72, Fe = 0.69, Ca = 0.46, Cu = 0.46, Mg = 0.34, K = −0.04. **b** *Axis1* Mg = −0.90, Ca = −0.88, Cu = 0.77, Zn = −0.69, Mn = 0.59, K = −0.37, Fe = −0.17. *Axis2* K = 0.73, Mn = 0.64, Cu = 0.41, Zn = 0.39, Mg = 0.27, Fe = 0.18, Ca = −0.14

The effect of preservation time on the earthworms and fly larvae was examined with respect to all the microelements analyzed in long and short exposures. In both species, the samples preserved in formaldehyde for long time periods clustered separately on the PCA plot from those exposed for short time periods (Fig. 3). This effect for alcohol is only visible in fly larvae (Fig. 3).

A two-way ANOVA performed on the scores from the first and second principal component axes provides a more detailed picture of the effect of different preservation methods on the elemental content of samples.

#### Earthworms

Along the first (horizontal) axis, a significant interaction was detected between the preservation mode and time (preservation *p* < 0.00001, time *p* < 0.00001, interaction *p* < 0.00001) (Fig. 3). Samples preserved in formaldehyde differed greatly from frozen samples in the content of Mg, K and Ca (variables with the highest loadings on the first axis). Among formaldehyde samples, a clear difference was visible between samples preserved for long and short times. Among the samples frozen and preserved in ethanol, there was no detectable effect of exposure time on elemental composition (Fig. 3). The second axis shows the differentiation of samples according to preservation method. The samples preserved in ethanol differed (higher concentration of Zn and Cu) from the samples preserved in formaldehyde as well as the frozen samples (preservation *p* < 0.00001, time ns, *p* = 0.05, interaction ns, *p* = 0.18).

#### Fly larvae

Samples preserved for long time periods differed from samples preserved for short time periods (first axis: preservation ns, *p* = 0.96, time *p* < 0.00001, interaction ns, *p* = 0.26; Fig. 3). Long-term preserved samples contained less Mg and Ca but more Cu. The second axis separates samples according to preservation method (preservation *p* < 0.002, time ns, *p* = 0.09, interaction *p* < 0.0007). Samples preserved in formaldehyde for long time periods had little K and Mn.

### The effect of preservation and drying methods on dry body mass

#### Earthworms

Before the experiments, the body masses of the earthworms did not significantly differ between experimental groups (*F*_5,33_ = 0.53, *p* = 0.75) (Table 4). During preservation, the earthworms lost their body mass, but the difference in the wet mass of animals preserved in ethanol and in formaldehyde was not significant (*t*_16_ = −1.7, *p* = 0.1) (Table 4). After drying, the dry body masses (% of fresh mass) of the earthworms preserved before drying were significantly lower than those frozen before drying in all experimental groups (Table 4). A two-way ANOVA indicated that there was an insignificant effect of drying method (*F*_1,33_ = 0.56 *p* = 0.46) and a highly significant effect of preservation method (*F*_2,33_ = 22.32, *p* < 0.0001). Frozen individuals differed from both the ethanol- and formaldehyde-preserved individuals (Tukey HSD *p* < 0.001), while no difference was observed between individuals preserved in ethanol and in formaldehyde (Tukey HSD *p* = 0.89). The interaction between the preservation method and drying method was insignificant (*F*_2,33_ = 1.20, *p* = 0.31).

**Table 4:** Fresh and dry mass (mean ± SE) of dried experimental animals that differ in the way they were preserved

	Initial fresh body mass (g ind^−1^)	Wet mass of preserved individuals (% of initial mass)	Dry mass (% of initial fresh mass)	
			Vacuum dried (50 °C)	Freeze-dried
Earthworms				
Frozen	1.2 ± 0.07		17.9 ± 0.9	16.3 ± 0.5
Preserved in ethanol	1.1 ± 0.09	58.6 ± 0.9	12.6 ± 0.6	13.0 ± 1.1
Preserved in formaldehyde	1.1 ± 0.07	55.9 ± 1.5	13.2 ± 0.9	13.2 ± 0.3
Fly larvae				
Frozen	0.09 ± 0.002		30.1 ± 0.7	29.2 ± 0.4
Preserved in ethanol	0.08 ± 0.002	93.8 ± 0.5	30.3 ± 0.5	30.5 ± 0.7
Preserved in formaldehyde	0.09 ± 0.003	98.6 ± 0.2	30.3 ± 1.3	29.3 ± 0.6

#### Fly larvae

Body mass loss during preservation was much lower in the fly larvae than in the earthworms (Table 4), but the difference between ethanol- and formaldehyde-preserved individuals was significant (*t*_26_ = 9.3, *p* < 0.0001). After drying, the fly larvae lost much less of their mass than the earthworms, and neither the preservation method nor the drying method affected the dry masses of individuals from all groups (two-way ANOVA: drying method *F*_1,39_ = 0.82, *p* = 0.37; preservation method *F*_2,39_ = 0.53, *p* = 0.59) (Table 4). The interaction between preservation method and drying method was insignificant (*F*_2,39_ = 0.34, *p* = 0.71).

## Discussion

The results of our study show that the use of solvents (ethanol or formaldehyde) in pitfall traps as killing agents and for preservation significantly affects the body composition and stoichiometry of earthworms, even during short exposure times. Insects (both adults and larvae) were affected only during a long (2 weeks) exposure; a 3-day exposure did not significantly change their chemical composition. Preservation time may play an important role in the migration of elements from animal bodies. When using pitfall traps to sample invertebrates for chemical analyses, the time of exposure must be reduced—collecting animals from the traps every day is recommended. The effect of preservation in ethanol on body composition appears to be weaker than that of formaldehyde. Drying of animals at low temperatures (50 °C) in a vacuum does not affect their body composition compared with freeze-drying.

The results of the present study are compatible with previous studies (Braun et al. 2009, 2012; Zödl and Wittmann 2003), although the methods and animals used were different. Among the animals studied, insects (both adults and larvae) appeared to be more resistant to the impact of preservation on body composition than oligochaetes. The short preservation time (3 days) did not significantly affect their element contents. The main factors determining the susceptibility of body chemistry to preserving liquids are the permeability of the body cover, the mobility of elements in the body tissues and the duration of exposure. Insects like arachnids are adapted to terrestrial environments, and their body cover prevents water loss in dry air, whereas oligochaetes (earthworms and enchytraeids), which inhabit the soil, escape dry conditions by moving into deeper soil layers or going into diapause (Edwards and Bohlen 1996; Lavelle and Spain 2005). Additionally, oligochaetes excrete high amounts of mucus, which allows them to keep their body moist even under dry environmental conditions. Another group of terrestrial animals that appears to lose elements quite easily during preservation is isopods, as has been shown by Zödl and Wittmann (2003). However, 2 weeks of exposure to formalin in pitfall traps affected the metal concentrations in only one (Zn) or two (Cu) of the four populations sampled by Zödl and Wittmann (2003).

Because of the differential permeability of the integument, animals preserved in ethanol or formaldehyde lose their body liquids at different rates. Because body liquids may contain certain amounts of elements (e.g., nitrogen, potassium, sodium and in oligochaetes) as well as products of nitrogen metabolism (containing urea, NH_3_, uric acid) that are excreted with mucus (Laverack 1963), their loss alters the chemical composition of their body. After 3 days of preservation, the earthworms lost over 40 % of their wet body mass, while the fly larvae lost less than 10 % (Table 4). The proportion of water lost during preservation in earthworms observed here is significantly higher than that lost in *Lumbricus terrestris* L. reported by Satchell (1971) and Raw (1962; cited after Satchell 1971). This inconsistency may be explained by the differences in water loss between species (Satchell 1971; Wetzel et al. 2005). Finally, the different proportion of the preservation agent relative to the mass of the preserved organism may provide another possible explanation.

The dry masses of the fly larvae did not differ significantly between treatment groups (frozen, preserved in ethanol or formaldehyde; Table 4) or between drying methods (freeze-dried vs. 50 °C vacuum-dried, Table 4). In contrast, the dry masses of the earthworms preserved in solvents were significantly lower than the dry masses of the frozen earthworms (Table 4). The body mass loss during preservation is mainly due to the excretion of mucus, a reaction to stress in earthworms. The excretion of mucus may affect the body composition of earthworms in two ways: some elements may be more condensed in the dry mass of particular tissues, while other elements may have lower concentrations as a result of the loss of mucus (Scheu 1991).

It is often difficult to judge which solvent—ethanol or formaldehyde—more strongly influences body composition (Figs. 1, 2; Table 3). The overall patterns in the changes in invertebrate body stoichiometry due to the exposure of preservation agents can be visualized using multidimensional analysis. The PCA plot on the two major axes suggests that the effect of formaldehyde is more pronounced (Fig. 3).

Another important factor is the time of exposure (Wetzel et al. 2005). For the insects studied (except cockroaches), during 2 weeks of preservation, potassium declined rapidly, as this element is very mobile and can be easily washed out, but the concentrations of other elements were not affected (Table 3). Three days of exposure did not result in any significant difference in potassium concentration in the fly larvae (Table 3). In contrast, the preservation of earthworms in both ethanol and formaldehyde resulted in significant changes in the concentration of almost all the elements studied, even after only 3 days of preservation. The potassium concentration in the earthworms preserved for 2 weeks in ethanol amounted to only 1/8 of those in the freeze-dried individuals, whereas the earthworms contained approximately 1/3 of the amount in the freeze-dried ones after 3 days of preservation in ethanol (Figs. 1, 2).

Our results show that preservation time plays an important role in the migration of elements from animal bodies. However, collecting insects for chemical analyses in pitfall traps filled with preservation solvent may be employed if the time of exposure is reduced to a minimum (collecting animals from the traps every day), but oligochaetes should be collected alive.

Our results suggest that there is no effect of drying method (vacuum in 50 °C and freeze-drying) on the results of invertebrate body composition analyses. Experiments by Zödl and Wittmann (2003) suggested that heat drying (at 105 °C) did not affect the concentration of heavy metals in invertebrate bodies; however, the authors noted that in the case of volatile elements (Hg, As) high temperature may result in erroneous estimations. Some authors have used even higher temperatures (70–130 °C, Alves et al. 2010; Braun et al. 2009; Jelaska et al. 2007; Visanuvimol and Bertram 2011). This may result in the evaporation of some organic compounds (e.g., lipids), resulting in the loss of dry mass and spuriously increasing the concentration of other elements. The high drying temperatures above the point of protein denaturation (approximately 70 °C) may cause a release of volatile forms of nitrogen; therefore, we recommend freeze-drying or heat-vacuum drying at lower temperatures.

## Conclusions

Our results show that the methods of handling animals before chemical analyses (sampling, pitfall traps, method of preservation, time of preservation and drying) may significantly affect results of the body composition and stoichiometry of invertebrates. However, the pitfall traps with an appropriate solvent may be used for sampling animals for chemical analyses if the exposition time is minimized. We recommend using ethanol as a solvent and exposition of traps limited to 1 day only.

## References

[CR1] Alves JM, Caliman A, Guariento RD, Figueiredo-Barros MP, Carneiro LS, Farjalla VF, Bozelli RL, Esteves FA (2010). Stoichiometry of benthic invertebrate nutrient recycling: interspecific variation and the role of body mass. Aquat Ecol.

[CR2] Bertram SM, Schade JD, Elser JJ (2006). Signalling and phosphorus: correlations between mate signalling effort and body elemental composition in crickets. Anim Behav.

[CR3] Bertram SM, Bowen MM, Kyle MM, Schade JD (2008). Extensive natural intraspecific variation in stoichiometric (C:N:P) composition in two terrestrial insect species. J Insect Sci.

[CR4] Braun M, Simon E, Fábián I, Tóthmérész B (2009). The effects of ethylene glycol and ethanol on the body mass and elemental composition of insects collected with pitfall traps. Chemosphere.

[CR5] Braun M, Simon E, Fábián I, Tóthmérész B (2012). Elemental analysis of pitfall-trapped insect samples: effects of ethylene glycol grades. Entomol Exp Appl.

[CR6] Couture JJ, Servi JS, Lindroth RL (2010). Increased nitrogen availability influences predator–prey interactions by altering host-plant quality. Chemoecology.

[CR7] Cross WF, Benstead JP, Rosemond AD, Wallace JB (2003). Consumer-resource stoichiometry in detritus-based streams. Ecol Lett.

[CR8] Duelli P, Obrist MK, Schmatz DR (1999). Biodiversity evaluation in agricultural landscapes: above-ground insects. Agr Ecosyst Environ.

[CR9] Edwards CA, Bohlen PJ (1996). Biology and ecology of earthworms.

[CR10] Edwards FK, Lauridsen RB, Armand L, Vincent HM, Jones JI (2009). The relationship between length, mass and preservation time for three species of freshwater leeches (Hirudinea). Fund Appl Limnol.

[CR11] Finke MD (2002). Complete nutrient composition of commercially raised invertebrates used as food for Insectivores. Zoo Biol.

[CR12] Finke MD (2007). Estimate of chitin in raw whole Insects. Zoo Biol.

[CR13] Finke MD, Winn D (2004). Insects and related arthropods: a nutritional primer for rehabilitators. J Wildlife Rehabil.

[CR14] Gibb TJ, Oseto TY (2006). Arthropod collection and identification: laboratory and field techniques.

[CR15] González AL, Fariña JE, Kay AD, Pinto R, Marquet PA (2011). Exploring patterns and mechanisms of interspecific and intraspecific variation in body elemental composition of desert consumers. Oikos.

[CR17] Hambäck PA, Gilbert J, Schneider K, Martinson HM, Kolb G, Fagan WF (2009). Effects of body size, trophic mode and larval habitat on Diptera stoichiometry: a regional comparison. Oikos.

[CR18] Jelaska LS, Blanuša M, Durbešić P, Jelaska SD (2007). Heavy metal concentrations in ground beetles, leaf litter, and soil of a forest ecosystem. Ecotox Environ Safe.

[CR19] Kagata H, Ohgushi T (2007). Carbon–nitrogen stoichiometry in the tritrophic food chain willow, leaf beetle, and predatory ladybird beetle. Ecol Res.

[CR20] Kay AD, Rostampour S, Sterner RW (2006). Ant stoichiometry: elemental homeostasis in stage-structured colonies. Funct Ecol.

[CR21] Knapp M (2012) Preservative fluid and storage conditions alter body mass estimation in a terrestrial insect. Entomol Exp Appl 143:185–190

[CR22] Larsen T, Ventura M, Damgaard C, Hobbie EA, Krogh PH (2009). Nutrient allocations and metabolism in two collembolans with contrasting reproduction and growth strategies. Funct Ecol.

[CR23] Larsen T, Ventura M, O’Brien DM, Magid J, Lomstein BA, Larsen J (2011). Contrasting effects of nitrogen limitation and amino acid imbalance on carbon and nitrogen turnover in three species of Collembola. Soil Biol Biochem.

[CR24] Lavelle P, Spain AV (2005). Soil ecology.

[CR25] Laverack MS (1963). The physiology of earthworms.

[CR26] Marichal R, Mathieu J, Couteaux MM, Mora P, Royc J, Lavelle P (2011). Earthworm and microbe response to litter and soils of tropical forest plantations with contrasting C:N:P stoichiometric ratios. Soil Biol Biochem.

[CR27] Nakamura K, Taira J (2005). Distribution of elements in the millipede, *Oxidus gracilis* C. L. Koch (Polydesmida: Paradoxosomatidae) and the relation to environmental habitats. Biometals.

[CR28] Nakamura K, Taira J, Higa Y (2005). Internal elements of the millipede, *Chamberlinius hualiensis* Wang (Polydesmida: Paradoxosomatidae). Appl Entomol Zool.

[CR29] Pokarzhevskii AD, van Straalen NM, Zaboev DP, Zaitsev AS (2003). Microbial links and element flows in nested detrital food-webs. Pedobiologia.

[CR30] Satchell JE (1971) Earthworms. In: Philipson J (ed.). Methods of study in quantitive soil ecology: population, production and energy flow. IBP Handbook, vol 18. Blackwel Scientific Publications, Oxford, pp 107–127

[CR31] Scheu S (1991). Mucus excretion and carbon turnover of endogeic earthworms. Biol Fert Soils.

[CR32] Spurgeon DJ, Weeks JM, Van Gestel CAM (2003) A summary of 11 years progress in earthworm ecotoxicology. Pedobiologia 47:588–606

[CR33] StatSoft Inc. (2011) STATISTICA (data analysis software system), version 10. http://www.statsoft.com

[CR34] Ter Braak CJF, Šmilauer P (2012) Canoco reference manual and user’s guide: software for ordination, version 5.0. Microcomputer Power, Ithaca, USA

[CR35] Villanueva VD, Albariño R, Canhoto C (2011). Detritivores feeding on poor quality food are more sensitive to increased temperatures. Hydrobiologia.

[CR36] Visanuvimol L, Bertram SM (2011). How dietary phosphorus availability during development influences condition and life history traits of the cricket, *Acheta domesticus*. J Insect Sci.

[CR37] Wetzel MA, Leuchs H, Koop JHE (2005). Preservation effects on wet weight, dry weight, and ash-free dry weight biomass estimates of four common estuarine macro-invertebrates: no difference between ethanol and formalin. Helgoland Mar Res.

[CR38] Woods HA, Fagan WF, Elser JJ, Harrison JF (2004). Allometric and phylogenetic variation in insect phosphorus content. Funct Ecol.

[CR39] Zödl B, Wittmann KJ (2003). Effects of sampling, preparation and defection on metal concentrations in selected invertebrates at urban sites. Chemosphere.

